# Delivery of Flavonoids and Saponins from Black Bean (*Phaseolus vulgaris*) Seed Coats Incorporated into Whole Wheat Bread

**DOI:** 10.3390/ijms17020222

**Published:** 2016-02-17

**Authors:** Rocio A. Chávez-Santoscoy, Marco A. Lazo-Vélez, Sergio O. Serna-Sáldivar, Janet A. Gutiérrez-Uribe

**Affiliations:** 1 Facultad de Ciencias Químicas e Ingeniería, Universidad Autónoma de Baja California—Campus Tijuana, Calzada Universidad 14418, Parque Industrial Internacional Tijuana, C.P. 22390 Tijuana, B.C., Mexico; ale.santoscoy@gmail.com; 2 Tecnológico de Monterrey, Campus Monterrey, Centro de Biotecnología FEMSA, Escuela de Ingeniería y Ciencias, Av. Eugenio Garza Sada 2501 Sur, C.P. 64849 Monterrey, N.L., México; marcoalazo@hotmail.com (M.A.L.-V.); jagu@itesm.mx (J.A.G.-U.)

**Keywords:** enriched foods, black bean seed coat, flavonoids, saponins, whole wheat bread, colon cancer

## Abstract

Cereal-based products can be used as vehicles for the delivery of relevant bioactive compounds since they are staple foods for most cultures throughout the world. The health promoting benefits of flavonoids and saponins contained in black bean seed coats have been previously described. In the present work, the effect of adding flavonoids and saponins from black bean seed coat to the typical yeast-leavened whole wheat bread formulation in terms of bread features, organoleptic properties and phytochemical profile was studied. The retention of bioactive compounds was determined and the inhibitory effects of *in vitro* enzyme digested samples on two colon cancer cell lines (Caco-2 and HT29) was evaluated. The addition of bioactive compounds did not significantly affect baking properties or texture parameters. Among organoleptic properties of enriched breads, only crumb color was affected by the addition of bioactive compounds. However, the use of whole wheat flour partially masked the effect on color. More than 90% of added flavonoids and saponins and 80% of anthocyanins were retained in bread after baking. However, saponins were reduced more than 50% after the *in vitro* enzyme digestion. The black bean seed coat phytochemicals recovered after *in vitro* enzyme digestion of enriched breads significantly reduced by 20% the viability of colon cancer cells without affecting standard fibroblast cells (*p* < 0.05).

## 1. Introduction

Nowadays, there is an increasing demand of health-promoting components in edible products. Functional foods are defined as innovative food products which can improve physical and mental well-being and prevent nutrition-related diseases of the consumer beyond basic nutrition [1]. The main strategy that has been followed to create functional foods is the addition of one or more components to improve a potential biological activity. These components may or may not be present in the original food. However, for successful design and development of functional food, it is necessary to provide special attention to the appearance and sensory properties of foodstuffs since, to the consumer, they are the more important attributes than the nutritional values.

Colorectal cancer is one of the leading causes of cancer deaths in the United States. Moreover, several epidemiological and laboratory studies suggest a relationship between colorectal cancer risk and dietary factors [2]. The development of safe and effective agents, such as functional foods, to reduce the risk of cancer and other chronic diseases is a high priority of health research. It has been reported that addition of spices, herbs and waste plant materials to enriched wheat bread can enhance its antioxidant properties [3]. Moreover, the enhancement of antioxidant properties and phenolic content of cereal-based foods such as durum spaghetti by the addition of buckwheat flour (Supreme) and bran (Farinetta) has been reported [4]. Particularly in colon cancer prevention, Reynoso-Camacho *et al.* [5] have reported that the consumption of tortillas, mainly from blue and white corns, significantly decreased adenocarcinoma incidence (up to 77.5%) in Sprague–Dawley Rats. Caco-2 and HT29 cell lines are the most common *in vitro* models to study the potential effect of bioactive compounds against colon cancer cells. However, there are some differences between these two cell lines: the human intestinal epithelial cell line Caco-2 forms a monolayer of absorptive enterocytes, while the HT29 cell line is an intestinal epithelial cell line which forms a monolayer of goblet cells [6]. In addition, the Caco-2 cell line is programmed to express lower levels of Cyclooxygenase-2 (COX-2) compared to HT-29 cell line; the overexpression of COX-2 provides to cells increased adhesiveness to the extracellular matrix and inhibition of apoptosis [7].

Herbal saponins were considered as potentially toxic in past years, because of their capability to hemolyse red blood cells; however, these phytochemicals have recently raised considerable interest for their health-promoting effects including antitumor, anti-inflammatory, cardiovascular, hepatoprotective, cholesterol-lowering and prebiotic-like effects on gut microbiota [8,9,10,11]. Meanwhile, flavonoids comprise the most common group of plant polyphenols and have been widely studied for their health benefits such as antioxidant, antiproliferative, hepatoprotective, and cardiovascular among others [12,13,14,15]. Additionally, these phytochemicals, saponins and flavonoids extracted from black bean (*Phaseolus vulgaris* L*.*), have been studied especially in terms of their antioxidant, anti-inflammatory [16,17,18], hypocholesterolemic effects in laboratory animals [19], and inhibitory effects in the absorption of lipids through micelle disruption and the overexpression of ATP-binding cassette (ABC) transporters such as ABCG5 and ABCG8 [11,19,20]. Therefore, black bean seed coats could be an efficient source of bioactive compounds that upon extraction can be supplemented in foods for the purpose of obtaining innovative products with additional health benefits. Likewise, flavonols and group B saponins have been efficient against hepatic and colon cancers [8]. Other authors have studied the inhibitory effect of water-soluble tannins from black bean on the proliferation of Caco-2 colon and MCF-7 and Hs578T breast cancer cells [21]. Thus, the supplementation of cereal-based products with bioactive compounds from black bean seed coats can improve the phytochemical profile and consequently exert health benefits, such as protection against oxidative stress and colon cancer.

Bread is considered the main staple food worldwide; it is consumed in practically every country around the globe because of its nutritive value, relatively low price and its simplicity of use. Bread is considered as an excellent source of energy, and an adequate source of protein and some essential minerals and other micronutrients. Thus, bread can be used as a vehicle for the delivery of bioactive compounds at suitable levels that provide health benefits for increased well-being [22,23,24,25]. However, the addition of bioactive compounds can be challenging because of their stability and the possible negative effect in dough functionality and bread features. There are a number of factors that may limit the effective enrichment of cereal products with bioactive compounds, such as instability of anthocyanins to light and heat and their susceptibility to degradative reactions [26]. Several authors have also reported that the incorporation of flavonoids and saponins could affect water solubility and viscosity [27,28]. In addition, previous reports evaluated the stability of saponins from soybean and chickpea during bread making and concluded that saponins were more stable after baking and were more bioaccessible in the yeast-leavened bread than in the original soybean or chickpea [29].

As a first approach, the objective of this research was to evaluate bread features and organoleptic properties of yeast-leavened whole wheat breads supplemented with flavonoids and saponins previously extracted from black bean seed coats. The retention of flavonoids and saponins in enriched breads after the bread making process and the effect on the reduction of viability of two colon cancer cells (HT-29 and Caco-2) lines exposed to *in vitro* digested bread extracts were studied.

## 2. Results and Discussion

### 2.1. Effect of Added Black Bean Seed Coat Extract on Baking Performance and Bread Features

There has been ongoing research showing that cooking beans generally lowers its antioxidant ability [30], furthermore cereal-based products are consumed as part of daily diet worldwide. Thus bread was used as a vehicle for the delivery of bioactive compounds from black bean seed coat. The weight, height, volume, color, and texture parameters of control bread and bread with black bean seed coat extract were evaluated (Table 1). The 0.5% addition of freeze-dried black bean seed coat extract (FDE) to whole wheat flour for bread making did not significantly affect baking properties. Several authors have reported that the incorporation of flavonoids and saponins to wheat dough reduced water solubility [27], bread specific gravity and apparent viscosity [28]. However, concentrations of saponins and flavonoids supplemented in the bread formulation used herein were small enough to not affect the properties of dough water absorption, apparent density and viscosity. Consequently, dough mixing time, bread height, weight and volume were not affected by the addition of FDE. Moreover, addition of 0.5% of the black bean seed coat extract did not significantly affect (*p* < 0.05) cohesiveness, hardness, chewiness and elasticity compared to control bread without the black bean seed coat extract (CN) (Table 1). Other authors have observed that the addition of antioxidant in dough did not affect cohesiveness, hardness and chewiness as much as the addition of fiber (pectin) [31]. Additionally, it was previously reported that the addition of other phenolic compounds, such as caffeic, ferulic and gallic acids did not affect the physical characteristics of resulting doughs or breads [32].

**Figure 1:**
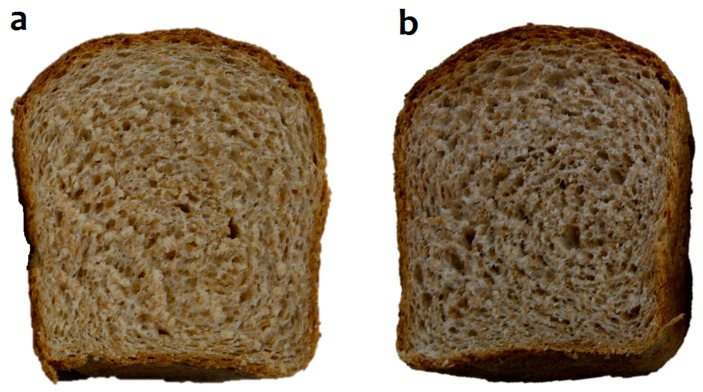
Appearance of (**a**) control whole wheat bread (CN); and (**b**) bread supplemented with 0.5% of black bean extract (BBE).

Sensory analysis and objective colorimeter values confirmed significant changes in color by changing a*, b* and L* values (Figure 1 and Table 1). The bread containing black bean seat coat extract (BBE) had lower L* (luminosity), b* (yellowness) and a* (greenness) values (Table 1). Changes in a*, b* and L* values were perceived in sensory analyses, since color was the only feature where the consumers detected a significant difference in bread samples. Other authors concluded that the addition of polyphenols to flour decreased L* values, thereby producing darker crumbs [33] and presence of anthocyanins significantly changed b* and a* values [34,35]. Since BBE was supplemented with polyphenols and anthocyanins, a significant change in a*, b* and L* values was expected, and why whole wheat flour was used for bread preparation. Consumers that performed sensory analyses did not notice significant differences in supplemented and control bread texture (*p* < 0.05), flavor (*p* < 0.05) and odor (*p* < 0.05). The organoleptic texture scores agreed with the objective texture properties determined with the texture analyzer (Table 1). As expected, the color of crumb was the only attribute that differed for the panelists (*p* < 0.05). Interestingly, the BBE had a significant better score in the 5-point hedonic scale, probably because panelists felt that the whole wheat bread was darker, as have been previously reported by other authors [36,37]. Finally, the overall acceptability of both breads (CN and BBE) was similar (*p* > 0.05) (Table 1). These results suggested that the use of whole wheat flour enriched with 0.5% of black bean seed coat extract for bread making was a good strategy to produce enriched breads without affecting organoleptic and physical bread properties, except for the bread crumb color feature (Table 1).

**Table 1:** Effect of the addition of black bean extract (FDE) on the physical characteristics of breads prepared with the straight-dough procedure.

Attribute	Control Bread (CN)	Bread with 0.5% Black Bean Extract (BBE)
**Baking Properties**		
Dough Water absorption (%)	70.13 ± 4.0	70.01 ± 3.1
Dough Mixing time (min)	4.79 ± 0.2	4.86 ± 0.2
Proof height (cm)	8.2 ± 0.1	8.2 ± 0.1
Bread height (cm)	8.4 ± 0.4	8.2 ± 0.1
Oven spring ^1^ (cm)	0.2 ± 0.3	0.0 ± 0.1
Bread weight (g)	151.5 ± 1.2	151.7 ± 2.5
Bread volume (cm^3^)	660.0 ± 38.5	673.3 ± 16.3
Apparent density (g/cm^3^)	0.229 ± 0.0	0.225 ± 0.0
**Crumb Color**		
a*	4.45 ± 0.51	2.81 ± 0.46 **^†^**
b*	20.39 ± 0.77	10.55 ± 0.46 **^†^**
L*	62.20 ± 2.09	52.42 ± 1.78 **^†^**
**Bread Crumb Texture Parameters ^4^**		
Cohesiveness (%)	87.0 ± 6.00	85.0 ± 5.00
Hardness (kg)	0.30 ± 0.25	0.27 ± 0.26
Chewiness	0.25 ± 0.19	0.22 ± 0.20
**Sensory Analyses ^2^**		
Color	3.28 ± 1.02	4.32 ± 1.00 **^†^**
Texture	3.81 ± 1.10	3.74 ± 1.08
Flavor	4.81 ± 1.23	4.83 ± 1.23
Odor	3.89 ± 1.47	4.01 ± 1.12
Overall acceptability	4.99 ± 1.02	4.97 ± 1.13

^1^ Oven spring = bread height − proof height; **^†^** Parameters were significantly different between CN and BBE (*p* < 0.05), Mean comparison was performed with a Student’s *t*-test. a*, b* and L*, are the components of color according to Comission Internationale de l´Éclairage (CIE), *a* axis green (−*a*) to red (+*a*), and the *b* axis from blue (−*b*) to yellow (+*b*) and the lightness represented by L. ^2^ Measured on the scale of 1 to 5 where 1 = dislike very much and 5 = like very much.

### 2.2. Quantification of Bioactive Compounds on Bread and Enzymatic Digested Samples

Between 88% and 91% of added flavonoids and saponins were retained in the BBE after baking (Table 2) while retention of total anthocyanins was 80%. This was greater than the retention of 79% observed in tortilla and 72% observed in gluten-free cookies both made with bioactive compounds from black bean seed coats [35]. This retention rate was attributed to the thermal stability of flavonoids from black bean [38]. Furthermore, it is well documented that the retention of other flavonoids, such as flavan-3-ols is adequate because they have good thermostability after baking [39,40,41]. Again, it has been reported that dry-heat (60 °C) did not have a significant effect on quercetin concentration, which is the major flavonoid associated with black beans [42]. On the other hand, it has been shown that bread ingredients could negatively affect the retention of bioactive compounds. However, Rosales-Soto *et al.* [43] concluded that anthocyanins and their antioxidant capacity in muffins were not affected by dough mixing time but they were partially diminished by baking. Also, Segev *et al.* [44] indicated that baking, frying and roasting colored chickpea seeds resulted in significantly higher levels of flavonoids in contrast with soaking and cooking, in which almost all flavonoids leached into the water. Therefore, results suggested that the addition of black bean coat extract into flour could be an excellent option to retain bioactive flavonoids in whole wheat or whole grain bakery products like bread, without affecting product characteristics. Results suggested that indeed, as it had been reported before, the changes in retention of bioactive compounds should not only be attributed to the thermal process (bread making process) but also to characteristics of the dough and other ingredients [35].

**Table 2:** Quantification of bioactive compounds in breads and enzymatically digested breads with and without the supplementation of black bean seed coat extract.

Bioactive Compounds in FDE ^1^	Bread		Enzymatically Digested Bread	
	Percentage of Retention %	BBE ^2^ mg/100 g·DW ^3^	Percentage of Retention %	BBE ^2^ mg/100 g·DW ^3^
	***Anthocyanin***			
**(mg cyanindin-3-glucoside equiv/100 g·DW ^3^)**	80.01 ± 1.20	31.28 ± 2.32	0.00	ND
	***Flavonoids***			
**Myricetin-3-*O*-glucoside**	89.21 ± 1.10	6.27 ± 1.23	88.17 ± 1.13	1.90 ± 0.02
**Quercetin-3-*O*-glucoside**	90.00 ± 1.10	56.10 ± 3.33	88.97 ± 1.21	16.28 ± 1.04
**Kaempferol-3-*O*-glucoside**	88.34 ± 0.41	0.396 ± 0.10	88.30 ± 0.50	0.12 ± 0.00
	***Saponins*^4^**			
**Phaseoside I**	90.20 ± 1.19	0.083 ± 0.06	0.00	ND *
**Soyasaponin Af**	91.87 ± 1.01	0.687 ± 0.28	49.32 ± 1.02	0.356 ± 0.40 *
**Deacetyl soyasaponin Af**	88.31 ± 2.14	0.103 ± 0.04	0.00	ND *
**Soyasaponin Ba**	89.97 ± 1.16	0.156 ± 0.04	0.00	ND *
**Soyasaponin αg**	91.01 ± 1.87	0.630 ± 0.09	48.42 ± 1.98	0.323 ± 0.07 *
**Soyasaponin βg**	89.71 ± 1.00	0.201 ± 0.03	49.41 ± 1.19	0.102 ± 0.03 *

^1^ FDE, Freeze-dried black bean seed coat extract; ^2^ BBE, Whole wheat bread with 0.5% of black bean seed coat extract; ^3^ DW, dry weight; ^4^ Concentration of saponins were significantly different in bread before and after enzymatic digestion (*). A One-way ANOVA test was performed and differences among means were compared at a level of significance of *p* < 0.05. ND, not detectable.

Among black bean phytochemicals, anthocyanins were the most affected phytochemical because of the heat used during the bread making process, since the percentage of retention was the lowest, and also because of the enzymatic digestion, as they were not detectable after the enzymatic digestion process. The sensibility of some anthocyanins to changes in pH and temperature has been previously reported [45,46], and in future experiments, the effect of thermal enzyme inactivation will be considered to evaluate the thermal degradation of black bean phytochemicals during enzyme digestion.

No significant losses of flavonols were observed after the *in vitro* enzymatic digestion of BBE (Table 2). These findings agree with Vallejo *et al.* [47] who found similar amounts of quercetin and kaempferol in broccoli (*Brassica oleracea*) before and after *in vitro* gastric digestion. As has been reported before, the effect of the proposed enzymatic digestion of bread was to release the antioxidant compounds retained by the polymeric matrix (like protein, bran, *etc.*) facilitating the extraction of soluble antioxidants [48]. In addition, an interaction between protein and added phenolics to enriched bread has been described; this could help to prevent phenolic degradation [22]. On the other hand, a reduction of 50% of saponins was observed in the enzyme digested BBE likely due to an alteration of the core structure of the aglycone. The reduction of saponins could be partially attributed to the thermosensibility of saponins from black bean seed coat reported before [11]. In addition, the detected reduction might be due to the bioconversion of the aglycone of saponins into other bioactive molecules, as has been reported before for red ginseng saponins after an *in vitro* gastric digestion [49]. According to previous investigations, the bioconversion route of saponins include deglycosylation followed by the modification of resulting aglycones by epimerization, and dehydration among other reactions [50]. These results do not necessarily indicate that the bioactivity has been reduced, since it has been reported that metabolites obtained after enzymatic digestion of saponins presented adequate bioactivity against gastro-intestinal cancer cells cultured *in vitro* [22,49,50,51].

### 2.3. Antiproliferative Activity of BBE against Colon Cancer Cells

**Figure 2:**
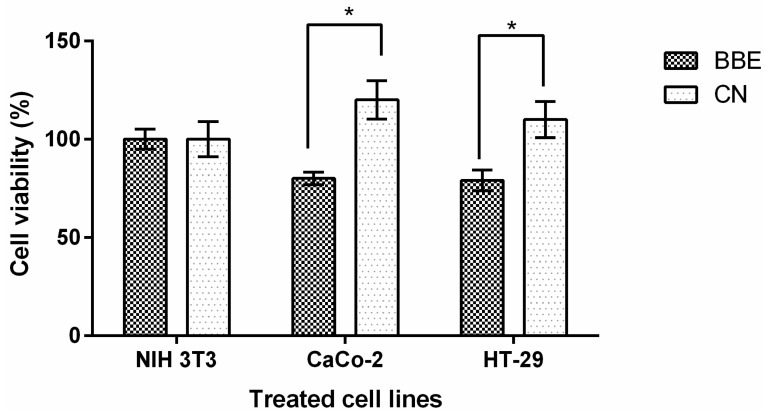
Growth inhibitory potency of whole wheat bread enriched with black bean seed coat extract (BBE) in comparison to whole wheat bread (CN) in cancer colon cell lines (HT-29, Caco-2) and standard fibroblast (NIH 3T3) used as control cell line. * Significant differences among treatments in each cell line after mean comparison with a Student’s *t*-test (*p* > 0.05). Treatments were carried out with the obtained supernatant of *in vitro* gastric digestion of 100 mg of bread (CN or BBE) diluted 1:10.

Treatment with digested BBE resulted in a significant reduction of viability of both Caco-2 and HT-29 cancer cells, with survival levels of 80.0% ± 3.20% (*p* < 0.05) and 79.5% ± 2.87% (*p* < 0.05), respectively (Figure 2) In addition, there were no significant effects on cell viability of regular fibroblasts (NIH 3T3) after treatment with extracts of digested BBE or CN breads (Figure 2), suggesting that the added bioactive compounds (flavonoids and saponins) to enriched bread decreased the viability of cancer cells without affecting regular fibroblasts used as control. These treatments included approximately 12.6 mg of initial bread/mL. In previous studies, the potential use of black bean methanolic extract as a source of compounds with anticancer activity has been shown [52]. *In vitro* results demonstrated the ability of black bean phytochemicals to induce apoptosis in HeLa cells and that this effect was not directly related with the antioxidant activity but with changes in the mitochondrial pathway [53]. Further experimentation is required to validate the *in vivo* effects in induced colon cancer animal models.

Extracts obtained from polyphenols rich sources also demonstrated significant effects when tested *in vitro*. In particular, bread supplemented with green coffee extract (1% contained 2.8 µM of chlorogenic acid per 100 g/L) increased the resistance of human colon (HT-29) and liver (HepG2) cells against oxidative H_2_O_2_, a source of oxidative stress [53]. Previous studies reported that concentrations between 0.3 and 0.8 mg/mL of a similar extract from black bean seed coat reduced colon and prostate cancer cell viability to 74% and 65%, respectively [8], without cytotoxicity towards normal cells. The results presented herein suggested that extracted flavonoids and saponins from black bean seed coat kept their bioactivity after the bread baking process and *in vitro* enzymatic digestion. Therefore, bread proved to be a good vehicle for supplementation of key bioactive compounds extracted from black bean seed coats, and thus, the addition of phenolic compounds to enriched breads might be an efficient chemoprotective strategy, as it has been reported before [3].

However, the *in vitro* digestion performed in the present work did not consider the possible absorption that might take place in the intestine. Additionally, it is important to point out that certain black bean phytochemicals have been related with the inhibition of α-amylase and α-glucosidase [54] and future research is required to evaluate this effect when the black bean seed coat extract is incorporated into processed foods. Further *in vivo* experiments should be conducted to verify the effect of enriched bread with flavonoids and saponins from black bean seed coat on colon cancer cells. Nevertheless, it has been reported that very small amounts of polyphenols are sufficient to significantly diminish cancer cell proliferation [55,56]. Interestingly, it has been reported that the combination of different polyphenols improved bioavailability, besides presenting a synergistic effect on their bioactivity [57,58]. Also, it has been reported that different types of phytochemicals associated to black bean seed coat tended to have a synergistic effect [11,19]. Thus, the addition of several compounds from black beans was more efficient strategy than if only the major bioactive compounds of the extract had been incorporated.

## 3. Experimental Section

### 3.1. Black Bean Seed Coat Extract

Whole black beans (*Phaseolus vulgaris* L. var.) were tempered in order to optimize the removal of the seed coats with mechanical decortication with abrasive disks [11]. The bioactive compounds were extracted at 27 °C from resulting ground seed coats with 60% ethanol in water (*v*/*v*) acidified with 0.1% of acetic acid using a mass-solvent ratio of 1:10 *w*/*v*. The mixture was stirred for 4 h at 250 rpm and left for one additional hour to allow sedimentation. The supernatant was recovered by vacuum filtering through Whatman paper No. 1. The resulting extracts were concentrated in a rotary evaporator to remove ethanol. The bath temperature was set at 50 °C, and pressure in the vacuum pump at a range of −70 to −90 kPa. Once ethanol was removed, the concentrated extract was lyophilized and the resulting freeze-dried powder stored at −80 °C until used. This freeze-dried powder of black bean seed coat extract (FDE) was used for the enrichment of wheat flour.

### 3.2. Bread Making

The pup loaf straight-dough bread micro-baking method 10–10.03 was utilized [59]. The formulations included composite flours containing 99.5% or 100% commercial whole wheat flour enriched with 0.5% or 0% of freeze-dried powder of black bean seed coat extract (FDE). Before mentioned formulations were called bread with black bean extract (BBE) and control bread (CN), respectively. The bakers formulation consisted of 5% refined cane sugar (Avance, Avance Comercial de Monterrey, Monterrey, N.L., Mexico), 4% vegetable shortening (Inca, Unilever de Mexico S.A de C.V.; Tultitlán, Edo. de Mexico, Mexico), 2% refined iodinated salt (La Fina, Sales del Istmo, Coatzacoalcos, Veracruz, Mexico), 2% dry yeast (*Saccharomyces cereviceae*) (Levadura Azteca, Iztapalapa, Mexico, D.F., Mexico), 2% dry milk (Nestle de Mexico, Mexico, D.F., Mexico) and 0.5% lecithin (Proveedores de Ingeniería Alimentaria S.A. de C.V. Monterrey, N.L., Mexico). All ingredients were incorporated in the initial mixing step. Doughs were mixed with the predetermined amount of 25 °C distilled water using a 100–200 g dough mixer (National Manufacturing Co.; Lincoln, Nebraska). Optimum water absorption and mix times were determined by observing dough properties or gluten development (film formation, gloss and dough stickiness). Resulting doughs were weighed and then cut into two identical pieces before placing them in a fermentation cabinet (National Manufacturing Co., Lincoln, Nebraska). Fermentation and baking conditions were made as previously reported [25]. Bake absorption, mixing time, proof height, loaf height, oven spring, loaf weight, loaf volume, and loaf apparent density were determined. Proof height and loaf height were determined with a proof height meter (National Manufacturing Co., Lincoln, Nebraska). The difference between these values was recorded as oven spring. Loaf volume was determined by rapeseed displacement (National Manufacturing Co.; Lincoln, Nebraska) according to method 10-05.01 of the AACC International [59]. Upon 30 min cooling at room temperature, BBE and CN breads were cut into 15 mm thick slices, packaged in sealed polyethylene bags and stored at room temperature for 24 h for sensory analysis. Several BBE and CN slices were freeze-dried (Freeze Drying 4.5, LABCONCO) at −50 °C, 0.036 mbar, for 48 h and stored at −80 °C until analysis.

### 3.3. Texture and Color of Bread Crumb

CN and BBE breads were objectively measured on three different slices (1.5 cm) with a colorimeter (CR 300, Minolta, Japan). Bread-crumb color parameters measured were L*, a*, b*. Crumb texture was evaluated with the texture analyzer (model TA.XT plus, Stable Micro systems, Godalming, Surrey, UK) with a trigger force of 0.048 N by bread firmness-compression test method 74-10.02 [60]. Cohesiveness, hardness, chewiness and adhesiveness were evaluated. Tests were conducted with one day old breads in the central part of the slice [25].

### 3.4. Sensory Analyses

In-house consumer panels (pilot consumer panels) made up of 40 consumers evaluated the sensory features and overall acceptability of control and experimental breads in individual booths after 24 h of baking [59]. Bread evaluation was performed in a sensory testing laboratory (ITESM-Campus Monterrey) according with the guidelines described before [61]. Each consumer was simultaneously given 2 coded samples along with a ballot, and was asked to rate color, texture, flavor, odor, and overall quality on a 5-point hedonic scale, where 1 was “dislike very much” and 5 was “like very much”.

### 3.5. In Vitro Digestion of Bread

Freeze-dried bread samples were ground and subjected to *in vitro* proteolytic digestion with the method recommended by Pasini *et al.* [62] with slight modifications with the aim of evaluating the effect on concentrations of key black bean phytochemicals and provide extracts for *in vitro* antiproliferative cancer studies. Briefly, 100 mg of ground bread samples (BBE or CN) were suspended in 4 mL of buffer pH 2.0 (50 mL 0.2 mol/L KCl, 13 mL 0.2 mol/L HCl, and water to make up 200 mL) and supplemented with 0.05 mg/mL of porcine pepsin P-7000 (Sigma-Aldrich, Toluca Edo. Mexico, Mexico) to emulate gastric protein hydrolysis. The digestion was carried out for 1 h at 37 °C in a shaking water bath (Hot-Shaker 7746-12110, Bellco Glass Inc., Vineland, NJ, USA) adjusted to a stirring speed of 28 rpm. Then, the pH of hydrolyzates was raised to 8 by the addition of boric acid buffer solution pH 9.1 (10 mM boric and acid, 50 mM NaOH to make up to 1) to which 0.25 mg/mL of pancreatin was added (Sigma-Aldrich, Toluca, Edo. Mexico, Mexico). The digestion was allowed to proceed for 120 min. The enzyme reaction was inactivated by placing hydrolyzates in a hot water bath adjusted to 80 °C for 2 min. After standing for 1 h, the samples were centrifuged for 10 min at 10,000× *g* and 4 °C (SL16R, ThermoScientific, Karlsruhe, Germany). The resulting supernatants were separated by filtration through a 0.45 mm sterile filter (Corning, New York, NY, USA) into 1.5 mL vials, which were immediately stored at −20 °C.

### 3.6. Sample Preparation for Quantification of Bioactive Compounds

Freeze-dried slices of the control (CN) or BBE bread were ground in a mill (Moulinex, DPA139) to pass through a 0.5 mm sieve to obtain powdered bread. Then, an extraction was performed as previously reported [35]. Briefly, 5 g of powdered samples of control (CN) or enriched (BBE) breads were extracted with 50 mL of 80% methanol in water (*v*/*v*). The mixture was stirred at 250 rpm for 3 h at 27 °C and finally left for one additional hour to allow sedimentation. The supernatant was recovered, concentrated, freeze-dried and finally dissolved in 1 mL of 80% methanol in water for the quantification of main bioactive compounds. Filtrated enzymatically digested samples were concentrated for analytical quantification of bioactive compounds. Every experiment was performed with three or five replicates.

### 3.7. Quantification of Bioactive Compounds in Extract, Bread and Enzymatically Digested Bread

Total anthocyanins were assayed according to the colorimetric method previously reported [35]. Flavonoids and saponins were quantified for FDE, BBE, CN and enzymatic digestions of BBE and CN through a High Pressure Liquid Chromatograph coupled with UV-Visible detector and an Evaporative Light Scattering Detector, HPLC-UV-VIS-ELSD (1200 Series, Agilent Technologies, Santa Clara, CA, USA), as previously reported [41]. The HPLC was equipped with a Zorbax SB-Aq (3 × 150 mm, 3.5 μm) column and data generated through the Agilent ChemStation software. The identification of flavonoids and saponins was confirmed with a HPLC-MS-TOF (Model G1969A Agilent 1100 Santa Clara, CA, USA), with the same chromatographic conditions and mass spectra were collected as previously reported [41]. The three main glycosylated forms of quercetin, myricetin and kaempferol were quantified with the chromatogram obtained at 360 nm using a calibration curve of the corresponding aglycone standard (Sigma, St. Louis, MO, USA). On the other hand, saponins were detected and quantified in a HPLC-ELSD detector using a calibration curve of soyasaponin I (Sigma, St. Louis, MO, USA) and expressed as equivalents in mg/100 g.

### 3.8. Effect of Enzyme Digested Bread Samples on Proliferative Activity of Colon Cancer Cells

Caco2 and HT29 cell lines (colon epithelial cancer cells) were maintained separately in DMEM-F12 and McCoy’s 5a Medium respectively, both cell media containing 10% of Fetal Bovine Serum (Gibco, Grand Island, NY, USA). Standard mouse embryo fibroblasts (NIH 3T3) were used as control and to determine if phytochemicals specifically affected colon cancer cell proliferation. The NIH 3T3 cells were also maintained in DMEM-F12 medium containing 10% Fetal Bovine Serum (Gibco, Grand Island, NY, USA). The method was performed according to Guajardo-Flores *et al.* [8]. Briefly, plates of 96-wells were prepared with 100 µL of a suspension containing 5 × 10^4^ cells/ml of each cell line at least 12 h before adding the filtrated supernatant of breads enzymatically digested as described before. Supernatants were previously diluted 1:10 with the respective medium for each cell line and then added to the cell suspension. After 48 h incubation, 20 µL of CellTiter 96^®^AQueous One Solution Cell Proliferation Assay (Promega, Madison, WI, USA) were added to each well in order to assess cell viability by measuring the absorbance at 490 nm in a microplate reader (Synergy HT, Bio-Tek, Winooski, VM, USA). Cell viability was calculated as percentage by the ratio between the absorbance obtained from cells treated with extracts and the absorbance obtained from untreated cells. All plates containing cells were incubated at 37 °C in a humidified 5% CO_2_ atmosphere.

### 3.9. Statistical Analysis

Results were expressed as means ± standard error. Data was analyzed with MINITAB 16 and JMP 11 software (SAS Corporation, Long Beach, CA, USA). The statistical analysis was performed by Student’s *t*-test when two groups were compared, while in experiments with more than 2 groups to compare, a one-way ANOVA followed by Tukey′s test to identify significant differences between treatments was performed. Differences among means were compared at a level of significance of *p* < 0.05. Every experiment was performed with three or five replicates.

## 4. Conclusions

In conclusion, our results suggested that the supplementation of bread with 0.5% FDE resulted in enhanced chemo-preventive *in vitro* properties in comparison with normal bread. Black bean seed coat enriched bread (BBE) contained significant amounts of flavonoids and saponins that inhibited *in vitro* growth of both colon cancer cell lines without affecting standard fibroblast cell viability. We showed that concentration of flavonoids and saponins decreased less than 90% during bread-making and that enzymatic digestion did not affect concentration of main flavonoids while saponins concentration was significantly reduced (*p* < 0.05). These findings might be due to the production of new metabolites formed during enzymatic digestion of saponins. Thus, the seed coat black bean extract rich in flavonoids and saponins had a positive bioactivity against two different colon cancer cell lines. Finally, the FDE enhanced the nutraceutical properties of breads produced by the straight dough making procedure. Results clearly indicated that FDE could be used as a food additive that could be incorporated (up to 0.5%) in the whole bread formulation without affecting baking performance and overall bread quality. Therefore, the black bean seed coat extract might be a value-added potential food ingredient for the preparation of yeast-leavened bakery and other related food products with improved functional and nutraceutical properties. The present results should not be considered definitive. It will be necessary to characterize these protective potentials further in animal models and human intervention studies.
